# Applying lessons from limb muscle disuse and ageing to better understand ventilator‐induced diaphragm dysfunction

**DOI:** 10.1113/EP092707

**Published:** 2025-07-10

**Authors:** P. H. C. Mesquita, J. L. Halle, J. D. Fuqua, B. F. Miller

**Affiliations:** ^1^ Aging and Metabolism Research Program Oklahoma Medical Research Foundation Oklahoma City Oklahoma USA; ^2^ The Oklahoma VA Medical Center Oklahoma Veterans Affair Oklahoma City Oklahoma USA

**Keywords:** proteostasis, recovery, unloading, VIDD

## Abstract

Mechanical ventilation (MV) is a life‐saving intervention applied to critically ill patients. A common consequence of MV is ventilator‐induced diaphragm dysfunction (VIDD), which is characterized by significant diaphragm atrophy and reduced contractile function. Older patients who receive MV are more likely to develop VIDD, have worse recovery, and higher mortality rates compared to younger adults. Despite the greater susceptibility of older adults to develop VIDD and lower survival rates compared to young adults, studies investigating the effects of ageing on VIDD and the recovery from MV are scarce. The field of limb skeletal muscle disuse has extensively shown that compared to adult limb muscles, aged limb muscles respond differently and have blunted recovery after disuse. This review summarizes the literature on the effects of MV on the diaphragm, discussing the available data on the effects of ageing and the recovery process after MV. We also provide an overview of the effects of ageing and the recovery of limb muscle from periods of disuse. We conclude with recommendations for future studies to apply lessons learned from the field of limb muscle disuse to the field of MV and VIDD.

## Abstract (highlights)

- **What is the topic of this review?**
  This review highlights the understudied area of diaphragm recovery after mechanical ventilation in aged individuals, and then reviews recovery from disuse atrophy in aged limb muscle for application to diaphragm recovery.
- **What advances does it highlight?**
  Almost all studies of the recovery of diaphragm after mechanical ventilation are on adult muscle, but we know from studies of aged limb muscle that recovery after disuse is not the same in adult and aged muscle. Therefore, this review highlights the need for studies of recovery from MV in aged diaphragm.

## 1 INTRODUCTION

Mechanical ventilation (MV) is an intervention used to support pulmonary gas exchange in critically ill patients. MV is commonly used in intensive care units (ICU) to save lives, but it often leads to a condition known as ventilator‐induced diaphragm dysfunction (VIDD) (Vassilakopoulos & Petrof, 2004). VIDD is characterized by diaphragm muscle atrophy and reduced contractile function (Powers et al., 2009; Vassilakopoulos & Petrof, 2004), which complicates weaning of patients off the ventilator and their subsequent recovery (Goligher et al., 2018). Older individuals account for 60% of ICU days (Angus et al., 2000), a percentage that has likely increased with the continued growth of the aged population since the publication of these data. Of all patients who receive MV, over 60% are above the age of 65 (Ma et al., 2021). Older patients who receive MV have higher hospital and 60‐day mortality rates compared to younger adults (<65 years) (Ma et al., 2021). The median survival after hospital discharge of people over 65 years old who received MV is less than 6 months (Ouchi et al., 2020). Therefore, most people who receive MV are over 65 and VIDD is particularly devastating in this population in terms of future health.

Over the past decades, several human and animal studies have investigated the effects of MV on diaphragm structure, function and the underlying mechanisms of VIDD. However, almost all these studies have been performed on young or adult models with few studies in aged models. In contrast, there are comparatively more studies of aged limb muscle and the impact of conditions such as muscle unloading. From these studies it is known that aged muscle is different from adult muscle and thus does not respond to conditions or interventions in the same way. The goal of this short review is to highlight the importance of studying MV in aged models, and to illustrate how what we have learned from aged limb muscle research could inform future studies of aged diaphragm.

## 2 IS A COMPARISON BETWEEN DIAPHRAGM AND LIMB MUSCLE APPROPRIATE?

First and foremost, both the diaphragm and limb muscles are skeletal muscles, which have highly structured contractile proteins, myofibrillar organization, connections to tendon to transmit force, similar energy substrate use, neural innervation and capillarization for blood perfusion, and most importantly, are plastic and adaptable. Given that the diaphragm is a highly specialized muscle, there are also some important considerations when comparing to limb muscle. While most limb muscles are recruited intermittently to generate movement, the diaphragm has a continuous and rhythmic activation. In humans, the fibre‐type composition of the diaphragm is similar to that of quadriceps muscle (∼45% type I, 55% type II) (Horwath et al., 2021; Levine et al., 2008). In rats, the fibre‐type composition of the diaphragm (∼35% type I, 35% type IIa, 30% type IIx/IIb) (Elliott et al., 2016) is more evenly distributed than most limb muscles (e.g., soleus: ∼88.8% type I, 11.2% type IIa; lateral gastrocnemius: ∼7.5% type I, 10.9% type IIa, 81.7% type IIx/IIb; plantaris: ∼6.9% type I, 27% type IIa, 66% type IIx/IIb) (Schenk et al., 2024). The diaphragm and limb muscles are both affected by ageing. For example, both the diaphragm and limb muscles show decreases in muscle size, force production and mitochondrial function with ageing (Brown et al., 2021; Elliott et al., 2016; Larsson et al., 2019), although the extent of these changes may vary. Important for this review is that in adult individuals, limb muscles and diaphragm seem to be affected by disuse in a similar manner, with both showing decreases in muscle mass and force production (Miller et al., 2019; Powers et al., 2009).

## 3 DISUSE AND RECOVERY OF LIMB MUSCLES

Limb skeletal muscles in humans and animals undergo rapid atrophy and contractile dysfunction in response to disuse. In healthy adults, limb muscle size and function decline non‐linearly with a rapid decline occurring during the first 1–2 weeks of disuse, followed by a plateau after ∼35 days (Marusic et al., 2021). In rodents, the degree of disuse atrophy differs between muscles, with the soleus muscle showing greater atrophy compared to some limb muscles. After 14 days of hindlimb unloading, the rat soleus muscle showed greater reduction in cross‐sectional area (CSA) of both type I (−60%) and type IIa (−59%) fibres compared to the tibialis anterior, which had no significant changes in type I or IIa, but decreased type IIb (−44%) fibres (Baehr et al., 2017). Human studies have also shown that the loss of muscle mass is variable across different muscle types. After 10–14 days of disuse, the loss of muscle fibre CSA ranged from 2.8% in the hip flexors, 5.0% in the quadriceps, 14.9% in the soleus, and ∼16.5% in the gastrocnemius muscles in healthy humans (Hardy et al., 2022). In studies using dry immersion as a model for human muscle disuse, the soleus showed up to 24% decrease in CSA after 21 days of disuse (Bokov et al., 2025). The greater disuse‐induced atrophy in the soleus muscle compared to other limb muscles may be due to its continuous recruitment for postural control under normal conditions, which could make it a useful comparison to diaphragm atrophy in response to MV. Muscle function declines more rapidly compared to the loss of muscle mass, ranging from −4% to −32% depending on the duration of disuse (Marusic et al., 2021).

In limb muscles, ageing does not appear to accelerate the loss of muscle mass during disuse (Baehr et al., 2016, 2017; Hodgson et al., 2022). In adult (9 months) and old (29 months) rats, the loss of muscle mass in response to 14 days of hindlimb unloading is variable across different limb muscles ranging from −9% to −38%, but not accelerated by age (Baehr et al., 2017). In humans, there are no overall differences in the rate of muscle atrophy between adult and old groups in response to 2 weeks of disuse (Hodgson et al., 2022). Regarding contractile function, two studies found similar decreases in force production between young (3–6 months) and old (23–25 months) rats after 14–21 days of hindlimb unloading (Mercier et al., 1999; Zhong et al., 2006). However, Baehr et al. (2016) showed a 17% reduction in maximum torque production in adult (9 month) and a 39% reduction in aged (29 months) rat muscle, despite having similar amounts of muscle mass loss in both groups. Both adult (10 months) and aged (30 months) rat muscles have reduced protein synthesis and increased protein degradation during hindlimb unloading (Lawrence et al., 2020; Miller et al., 2018).

Despite adult and aged individuals having similar rates of atrophy during disuse, the regain of muscle mass and function after reloading is impaired in aged compared to adult individuals. Older humans showed incomplete recovery of muscle mass and function after 14 days of rehabilitation following 14 days of bedrest, while adult counterparts had complete recovery (Pisot et al., 2016). The current state of this field is that this inability to regain muscle mass after disuse is because of deficiencies in protein synthesis and impaired responses to stimuli that increase protein synthesis (i.e., anabolic resistance; McKendry et al., 2021). Our recent studies do not support that protein synthesis is deficient during recovery from disuse since our data show that protein synthesis is higher in aged muscle compared to adult muscle (Fuqua et al., 2023; Miller et al., 2019). These higher rates are further supported by higher rates of ribosomal biogenesis and mammalian target of rapamycin (mTOR) signalling (Fuqua et al., 2023; Miller et al., 2019). As expanded upon below, these findings are a key lesson learned in our studies of recovery after disuse atrophy.

## 4 EFFECTS OF DISUSE ON DIAPHRAGM

Although different types of MV are currently available, we focus on full support positive‐pressure MV, which is most studied, during which the ventilator provides all the ventilation to the lungs and the respiratory muscles are inactive. An important point is that the diaphragm is inactive during MV, which is like disuse in the limb muscle. Due to the innate challenges and limitations to study the effects of MV in humans, the field has relied heavily on animal models to investigate VIDD. In most adult (∼4–6 months) animal studies, myofibre CSA decreases in all fibre types (from 18% to 36%) within 12 h of MV (Hyatt et al., 2021). Similarly, MV results in a rapid reduction in diaphragm submaximal and maximal force production, ranging from 15% to 50% reduction in maximal specific force production (Powers et al., 2009; Vassilakopoulos & Petrof, 2004). In human studies, MV leads to rapid atrophy (>50% after 18–69 h of MV) of both type I and type II diaphragm muscle fibres (Levine et al., 2008) and a decline in force production (Jaber et al., 2011). The decline of diaphragm muscle mass and force production seems to follow a progressive trajectory, with greater declines observed with longer duration of MV (Corpeno et al., 2014; Grosu et al., 2012).

**FIGURE 1:**
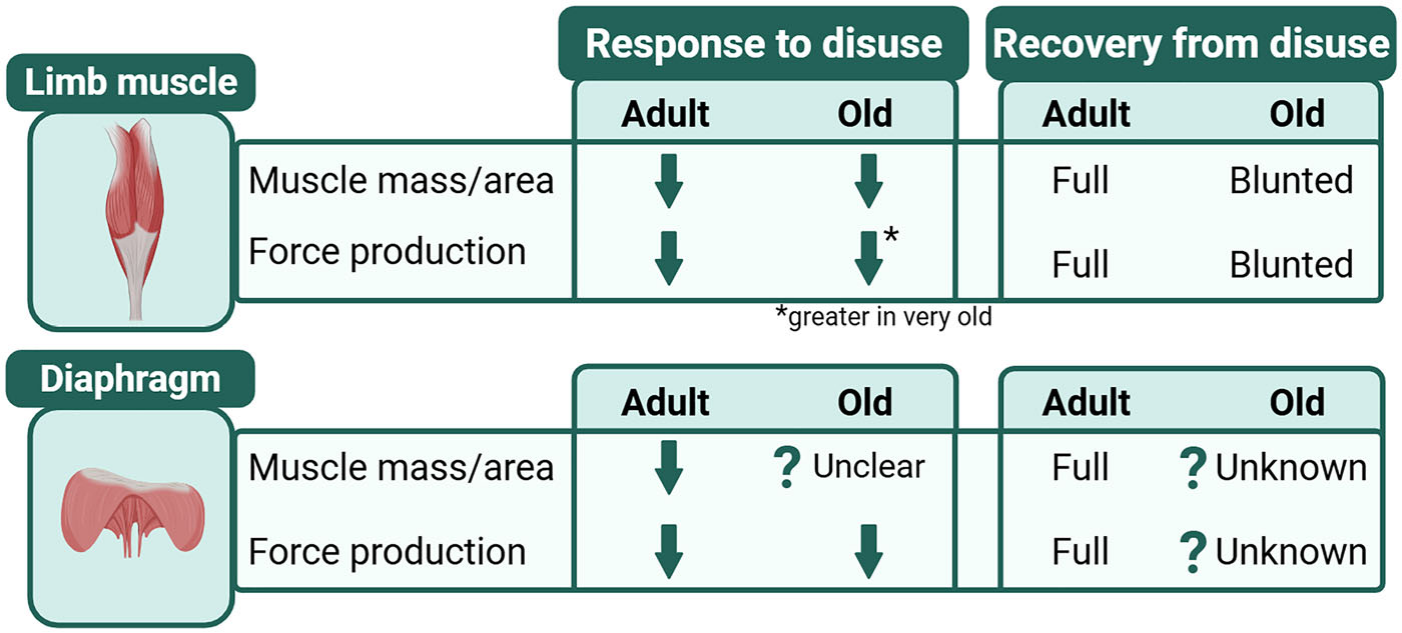
Response and recovery of adult and aged limb muscles and diaphragm following disuse.

The mechanisms leading to the decline in muscle mass and force production in response to disuse are similar between diaphragm and limb muscles. Compared to non‐atrophy conditions, the diaphragm shows decreased protein synthesis (Hudson et al., 2015) and increased protein degradation (Hyatt et al., 2021). Although previous studies have examined changes in protein synthesis and degradation in different limb muscles (e.g. soleus vs. tibialis anterior) during disuse (Baehr et al., 2017), they primarily relied on puromycin incorporation and indirect markers of protein degradation – methods with notable limitations. To address these challenges, we have developed approaches that employ long‐term deuterium labelling combined with non‐steady‐state equations to quantify synthesis and degradation rates (Kobak et al., 2021). However, studies using these methods to investigate the response of different limb muscles to disuse are still needed. Besides changes in protein synthesis and degradation, like limb muscles the diaphragm shows increased oxidative stress (Morton et al., 2019), altered lipid composition (Picard et al., 2012) and disruption of sarcomeres (Jaber et al., 2011) after a period of disuse. Impaired mitochondrial function is also commonly observed in the diaphragm and limb muscles after disuse (Picard et al., 2015; Powers et al., 2012). However, findings in limb muscles are not entirely consistent and may depend on the duration of the disuse, as some studies have reported no significant changes in mitochondrial function following disuse (Zuccarelli et al., 2021). Collectively, the available literature shows that in adult individuals, diaphragm and limb muscles respond similarly to disuse, with rapid and progressive atrophy and contractile dysfunction (Figure 1), and similar changes in molecular processes.

An important point is that while both diaphragm and limb muscles seem to respond similarly to disuse, the context in which the disuse occurs can significantly influence the results. Under certain disease conditions and stays in the ICU, both the limb muscle and the diaphragm will encounter a similar systemic environment that is marked by inflammation. However, under other conditions such as elective surgery, limb disuse may not have a complicated systemic environment. Currently, most studies in rodents are under otherwise healthy conditions, and thus incorporation of manipulations that change the systemic environment to those seen in the ICU or disease conditions are still warranted.

The importance of considering age when studying the effects of MV on diaphragm was highlighted over 10 years ago in a review paper by Powers et al. (2013), but little progress has been made to explore the effects of age and its impact on the diaphragm response to MV. In a recent review by Powers et al., 60 studies using animal models were cited, but none of the studies used aged models (Powers, 2024). In the first animal study to investigate the effects of age on VIDD, Criswell et al. (2003) found similar decreases in diaphragm specific force in young (4 months) and old rats (30 months) subjected to 12 h of MV. Single diaphragm muscle fibres also show declines in force production between adult (7–8 months) and old (28–32 months) rats after 5 days of MV (Cacciani et al., 2014; Ogilvie et al., 2016). On the other hand, Ogilvie et al. (2016) showed that administration of a heat‐shock protein chaperone co‐inducer during MV partially restored diaphragm force production in adult, but not in old rats, although with only two to three rats in each group. In a pilot study, Lyu et al. (2021) showed significant age‐related differences in the diaphragm response to 5 days of MV. Using both transcriptomics and proteomics, the authors found that the diaphragm of adult (7–8 months) rats showed decreases in processes related to energy metabolism, while old (28–32 months) rats primarily had increased immune and inflammation responses to MV. It is not known if, like limb muscles, adult and aged diaphragm have similar changes in protein synthesis and degradation or if aged diaphragm has disrupted proteostatic maintenance after MV.

Few studies have investigated recovery of the diaphragm after MV. Benli et al. (2024) reported no changes in diaphragm function of patients 5 days after extubation. Another study observed no changes in diaphragm thickness in mechanically ventilated COVID survivors 3 or 6 months after extubation, although inspiratory capacity increased from 3 to 6 months (Nunez‐Seisdedos et al., 2023). It is important to note that studies assessing diaphragm recovery in human patients may be biased toward patients who either did not develop VIDD or experienced milder effects. Animal studies on the recovery of the diaphragm after MV are also limited (Table 1), partially due to technical challenges with weaning animals from the ventilator. In the first animal study investigating the reloading of diaphragm after MV, Van Gammeren et al. (2005) showed no recovery of diaphragm force production after 2 h of spontaneous breathing (i.e., regular unassisted breathing) after MV. Thomas et al. (2013) showed that after as little as 3 h of recovery, the diaphragm had increased maximal force, fibre type IIx/IIb CSA, and activation of the mTOR signalling pathway compared to immediately after MV, while there were no changes in protein degradation and oxidative damage. The authors concluded that there was a full recovery of the diaphragm after 4–7 h of spontaneous breathing after MV. However, it is important to note that there was no control group in the study of Thomas et al., such that even though improvements in diaphragm function were found, it is not known the extent of the recovery compared to a control group or baseline values. Bruells et al. (2014) showed that diaphragm force production remained significantly lower after 12 h of recovery compared to controls but returned to control levels after 24 h. Markers of oxidative damage, anabolic signalling and proteolysis returned to control levels after 24 h of recovery. Dridi et al. (2020) found that even though diaphragm force production was restored after 24 h of recovery after MV in a mouse model, a reduction in force production was found 10 days after MV. This delayed deleterious effect of MV was related to changes in type 1 Ca^2+^ release channel/ryanodine receptor 1 remodelling. All the abovementioned studies were done in young/adult animals.

**TABLE 1:** Summary of studies investigating the recovery of the diaphragm after mechanical ventilation.

Study	Sample/MV duration	Recovery method/duration	Main Results
Van Gammeren et al. (2005)	Sprague–Dawley rats, 4 months old, female, 24 h of MV	Spontaneous breathing (under anaesthesia), 2 h	Force production remained low after 2 h of recovery
Thomas et al. (2013)	Wistar rats, adult (age not reported), male, 24 h of MV	Spontaneous breathing (under anaesthesia), 1, 2, 3, and 4–7 h	Higher force production at 3 and 4–7 h of recovery Greater fibre type IIx/b CSA at 3 and 4–7 h Higher mTOR signalling at 3 and 4–7 h No changes in markers of protein degradation and oxidative damage
Bruells et al. (2014)	Adult rats (strain, age, or sex not reported), 12 h of MV	Spontaneous breathing (Extubated and fully awake), 12 and 24 h	Force production remained low at 12 h but recovered at 24 h Markers of oxidative damage, anabolic signalling and proteolysis returned to control levels after 24 h of recovery
Dridi et al. (2020)	C57BL/6 mice, 12 weeks old, male, 6 h of MV	Spontaneous breathing (extubated and fully awake)^*^, 1 and 10 days	Force production recovered at 1 day but was lower 10 days after MV Type 1 Ca^2+^ release channel/ryanodine receptor 1 remodelling after MV and maintained at 1 day and 10 days of recovery

* Method for recovery not reported but was inferred based on the discussion. Abbreviation: MV, mechanical ventilation.

In summary, adult diaphragm, like adult limb muscle, has rapid declines in mass and function with disuse. Like adult limb muscle, adult diaphragm recovers after a period of disuse. However, for both limb and diaphragm muscle, there are impairments with age. In limb muscle, these impairments with age do not necessarily impact the degree of loss of mass but do have greater losses of function. Further, there is a failure to completely regain mass and function with reloading after a period of disuse. By comparison, there are relatively few studies on the degree of loss with MV in aged diaphragm, and many unknowns about recovery of aged diaphragm when voluntary breathing is reintroduced (i.e., reloading). This lack of knowledge creates a gap in the ability to treat recovery from VIDD. However, given the similarities between limb and diaphragm muscle, lessons from recovery of aged limb muscle could potentially provide a road map for recovery of aged diaphragm from VIDD.

## 5 NEW LESSONS LEARNED FROM LIMB MUSCLE TO APPLY TO DIAPHRAGM

As mentioned previously in the review, it is thought that there is an anabolic resistance of aged muscle that impairs recovery. This focus has led to efforts to aid recovery by targeting anabolic pathways, particularly the activation of mTOR complex I (mTORC1), since this activation increases protein synthesis and decreases protein breakdown. What is often overlooked is that studies that have compared adult and aged muscle show that mTORC1 signalling and protein synthesis are already higher in aged limb muscle compared to adult at baseline and during reloading (Baehr et al., 2016, 2017; Fuqua et al., 2023; Miller et al., 2019). Further, meta‐analyses show that strategies that activate mTOR, like protein supplementation (Yoshimura et al., 2025), lack efficacy in increasing muscle mass and/or function in aged muscle. The question then becomes why does limb muscle fail to regrow if anabolism is not deficient?

**FIGURE 2:**
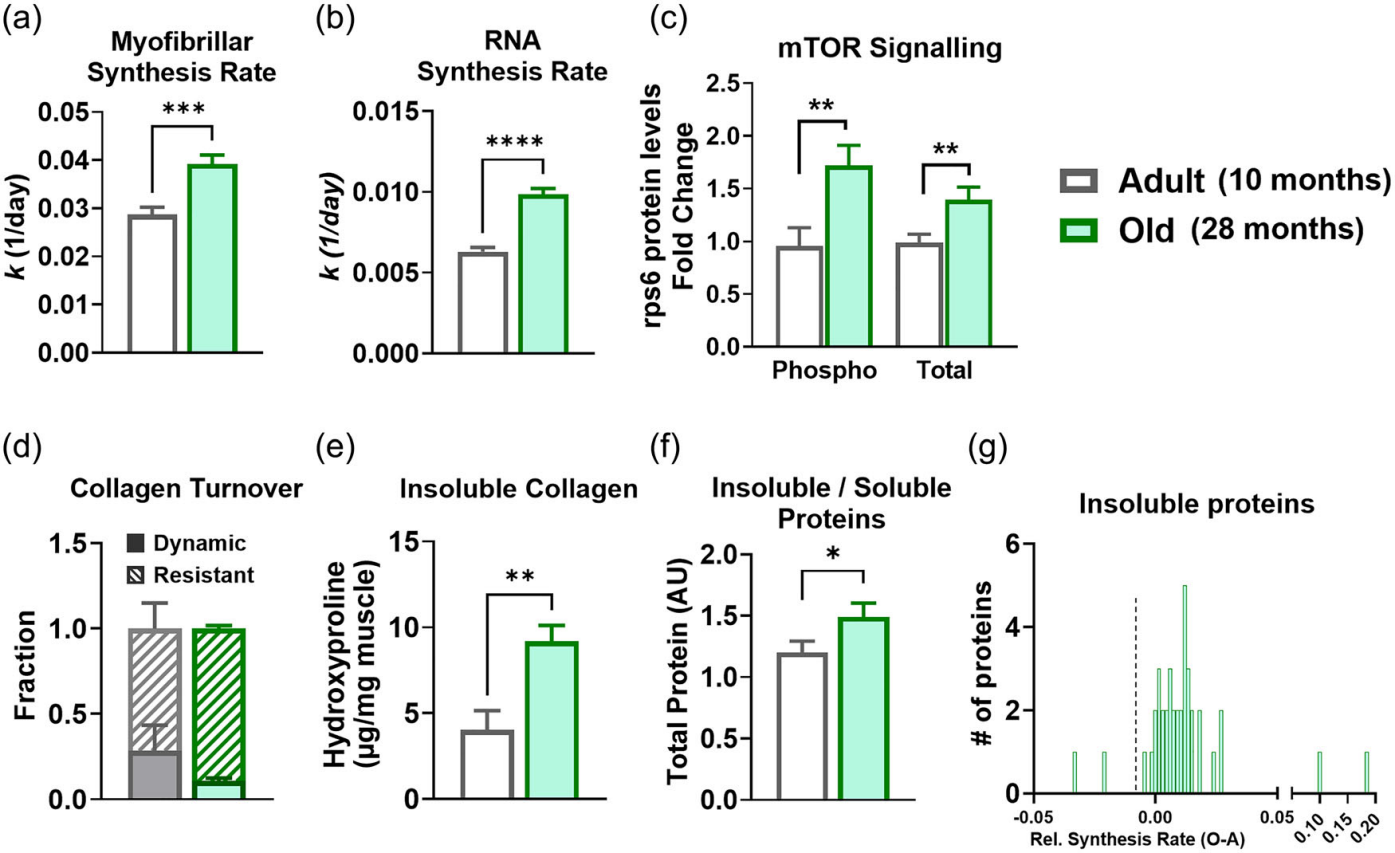
Blunted recovery of aged limb muscles following disuse is not driven by impaired protein synthesis but is associated with increased collagen deposition and insoluble and aggregate proteins, indicating compromised proteostatic maintenance. (a) Synthesis rates of myofibrillar proteins. (b) RNA synthesis rates. (c) Protein levels of phospho‐ribosomal protein S6 (rpS6) and total rpS6. (d) Fraction of collagen pool that is dynamic or actively turning over (filled bars) and resistant to turnover (striped bars). (e) Insoluble collagen content determined by hydroxyproline assay. (f) Ratio of detergent insoluble to detergent soluble proteins. (g) Relative synthesis rates (old – adult) of individual insoluble proteins. (a, b, c, f, g) show data obtained from the gastrocnemius muscles, while (d, e) show data obtained from the soleus muscles from adult (10 months) and old (28 months) male Fisher 344/BN rats during recovery after hindlimb unloading. Data adapted from Fuqua et al. (2023) – https://creativecommons.org/licenses/by‐nc‐nd/4.0/.

In contrast to previous studies, our studies show that the higher anabolism in aged muscle compared to adult muscle leads to a failure to maintain proteostasis. We subjected adult (10‐month) and old (28‐month) male rats to a 14‐day period of hindlimb unloading followed by deuterium oxide (D_2_O) labelling during reloading up to 60 days (Fuqua et al., 2023). We repeated the finding that ribosomal RNA (a measure of ribosomal biogenesis) and myofibrillar protein synthesis rates are higher in old muscle compared to adult muscle over a period of reloading. The higher rate of RNA synthesis is a reliable indicator of mTOR activity, and we confirmed that there was higher mTOR activity in the aged compared to adult rats (Fuqua et al., 2023). Despite these higher values, after 60 days of recovery old muscle failed to fully recover from disuse atrophy while adult muscle recovered by 15 days. We found that compared to adult muscle, old muscle had a lower proportion of collagen that is actively turning over and a greater proportion that is resistant to turnover, which likely led to increased insoluble collagen content. In addition, we found that old muscle accumulated insoluble proteins, which are enriched with protein aggregates, to a greater degree than adult muscle, indicating impaired proteostasis (Fuqua et al., 2023). We analysed the insoluble protein fraction and found that there was an accumulation of proteins tagged with p62, a marker of autophagy, and lysine‐48 (K48), a marker of ubiquitination, which was higher in old compared to adult muscle (Fuqua et al., 2023). When we performed proteomic analyses on the protein aggregates, we found that at the end of hindlimb unloading in aged muscle, 18 of the top 20 most abundant proteins were myofibrillar, while after reloading, 16 of the top 20 most abundant proteins were myofibrillar. We found that contractile proteins represented 89.2% and 84.5% of the protein abundance in the PAs. By taking advantage of our D_2_O labelling we showed that for proteins represented in the insoluble protein fraction of both adult and aged muscle the rates of synthesis were clearly higher in old muscle compared to adult muscle. In short, our study indicates that the increased anabolism in aged limb muscle resulted in synthesis rates that exceeded the capacity for proper protein processing, likely impairing breakdown, so that collagen, insoluble and aggregate proteins accumulated (Figure 2).

These observations from limb muscles beg the question of whether there is a failure to maintain proteostasis in aged diaphragm during MV, which contributes to VIDD during recovery. At this point, these studies do not exist. There are several questions that should be answered, like: Is mTORC1 activity high in aged diaphragm? What are the rates of protein synthesis and breakdown in diaphragm during MV or after reloading? And do protein aggregates accumulate in aged diaphragm during MV or reloading? It is reasonable to hypothesize that aged diaphragm will not recover in the same manner as young/adult diaphragm and that strategies for VIDD recovery for aged diaphragm should focus on treatments that maintain proteostasis. Current candidates for such treatments include the mTOR inhibitor rapamycin or autophagy activators.

## 6 CONCLUSIONS AND FUTURE DIRECTIONS

Mechanical ventilation is a lifesaving procedure used for patients in ICUs. However, MV commonly leads to VIDD, which compromises recovery from MV and survival. The outcomes and mechanisms of VIDD have been extensively studied, but the field has relied almost exclusively on young/adult animals. This lack of studies in aged models is a significant gap in the literature since we know from studies in limb muscle that adult and aged muscle have different mechanisms of atrophy and recovery. Of particular interest to us, is the lack of recovery in limb muscle and how this may be similar in diaphragm. We have focused on recovery since it appears to be more amendable, mechanistically and practically, to therapeutic interventions compared to the disuse period. Future studies should investigate how ageing affects the response and the recovery of the diaphragm from MV. Based on the field of limb muscle disuse, protein degradation, aggregation and extracellular matrix are aspects that deserve attention and could be the focus of future studies. Only after we understand the differences in the response of adult and aged diaphragm to MV will we be able to develop therapies to improve the recovery and survival of older patients subjected to MV.

## AUTHOR CONTRIBUTIONS

All authors have read and approved the final version of this manuscript and agree to be accountable for all aspects of the work in ensuring that questions related to the accuracy or integrity of any part of the work are appropriately investigated and resolved. All persons designated as authors qualify for authorship, and all those who qualify for authorship are listed.

## CONFLICT OF INTEREST

None declared.
